# Transglutaminase Cross-Linked Gelatin-Alginate-Antibacterial Hydrogel as the Drug Delivery-Coatings for Implant-Related Infections

**DOI:** 10.3390/polym13030414

**Published:** 2021-01-28

**Authors:** Chung-Kai Sun, Cherng-Jyh Ke, Yi-Wen Lin, Feng-Huei Lin, Tung-Hu Tsai, Jui-Sheng Sun

**Affiliations:** 1 Institute of Traditional Medicine, School of Medicine, National Yang Ming Chiao Tung University, No. 155, Sec. 2, Linong Street, Taipei 11221, Taiwan; samcksun@gmail.com or; 2 Biomaterials Translational Research Center, China Medical University Hospital, No. 2, Yude Rd., Taichung City 40447, Taiwan; fonchanwd@gmail.com; 3 Institute of Biomedical Engineering, College of Medicine, National Taiwan University, No. 1, Sec. 4, Roosevelt Rd., Taipei 10617, Taiwan; zhew520@gmail.com (Y.-W.L.); double@ntu.edu.tw (F.-H.L.); 4 Institute of Biomedical Engineering, College of Engineering, National Taiwan University, No. 1, Sec. 4, Roosevelt Rd., Taipei 10617, Taiwan; 5 Division of Biomedical Engineering and Nanomedicine Research, National Health Research Institutes, No. 35, Keyan Road, Zhunan, Miaoli County 35053, Taiwan; 6 Department of Orthopedic Surgery, College of Medicine, China Medical University, No. 2, Yu-Der Rd., Taichung City 40447, Taiwan; 7 Department of Orthopedic Surgery, National Taiwan University Hospital, No. 7, Chung-Shan South Road, Taipei 10002, Taiwan

**Keywords:** transglutaminase, gelatin-alginate hydrogel, implant-related infections, antibiotic, gentamicin, vancomycin, drug delivery

## Abstract

Implant-related infection may be catastrophic and result in poor functional outcome, chronic osteomyelitis, implant failure or even sepsis and death. Based on a transglutaminase (TGase) cross-linked/antibiotics-encapsulated gelatin-alginate hydrogel, the main aim of this study is to establish an effective antibiotic slow-release system. The second aim is to evaluate the efficacy of a hydrogel-encapsulated antibiotic-containing titanium pin in preventing implant-related infections in a rat model. The prepared gelatin/alginate/gentamicin or vancomycin hydrogel was covalently cross-linked with transglutaminase (TGase). Its drug release profile and cytotoxicity were determined and the Wistar rat animal model was performed to validate its efficacy by radiographic examination, Micro-CT (computed tomography) evaluation and histo-morphological analysis at 12 weeks after surgery. When gelatin and alginate were thoroughly mixed with TGase, both 0.5% and 1.0% TGase can effectively cross link the hydrogel; the release of antibiotic is slowed down with higher degree of TGase concentration (from 20 min to more than 120 h). In the animal study, antibiotic-impregnated hydrogel is effective in alleviating the implant-related infections. Relative to that of a positive control group, the experimental group (vancomycin treatment group) showed significant higher bone volume, more intact bony structure with only mild inflammatory cell infiltration. This newly designed hydrogel can effectively deliver antibiotics to reduce bacterial colonization and biofilm formation on the implant surface. The remaining challenges will be to confer different potent antibacterial medications with good biocompatibility and fulfill the safety, practical and economic criteria for future clinical translation.

## 1. Introduction

The implanted biomaterials play a key role in the success of current orthopedic procedures, but one of the major complications is the development of infection. The current global infection risk is 2–5% [1] and one of the most challenging complications is infection after the implantation of medical device [2]. Implant-related infection may be catastrophic and result in implant failure, chronic osteomyelitis, poor functional outcome or even sepsis and death [3,4,5,6]. 

When bacterial strains adhere to the surface of foreign materials and implants, they can proliferate and produce biofilm, and then even cause peri-implant osteomyelitis; thereby reducing susceptibility to antibiotics. It is then complicated and difficult to treat this kind of infection. The biofilms act as an impenetrable mechanical barrier against soluble agents, then systemic antibiotics can not overcome this limit to treat or prevent the biofilm-related infections [7].

Recent progress of the drug delivery has inspired further progress of biopolymer composites with a tailored mechanical property and stable characteristics for pharmaceutical and biomedical applications. Numerous natural biopolymer composites have been exploited in recent years to improve their physical and chemical properties and add new functionalities. Natural polymer-based hydrogels, such as chitosan and alginate, are both biocompatible and biodegradable and have inherently low immunogenicity, which makes them suitable for physiological drug delivery [8,9]. Sodium alginate mixed with gelatin (Alg-Gel hydrogel) has been widely used in tissue engineering. However, scientists have validated that changing properties (viscosity, stiffness) of hydrogel will significantly impact cell behavior and tissue formation [10]. Microbial transglutaminase is a member of the transglutaminases enzymes family, it is thermostable (in the range of 0–60 °C) with an optimal temperature to catalyze cross-links between protein molecules at 50 °C to 55 °C [11]. Used to produce site-specific protein conjugates derivatized at the level of Gln and/or Lys residues, microbial transglutaminase (mTG) has been successfully applied to different biotechnologies [12]. The cross-linked hydrogels can potentially be used for the development of “smart” delivery systems, which are capable of control release of the encapsulated drug at the targeted site. 

Implant-related infections can lead to high medical expenditure and be catastrophic for patients. Traditional treatments for implant-related infections include intravenous antibiotics, repeated radical debridement, and replacement of internal fixators. However, the prevention is more important than to treat it. Different treatment strategies have been adopted to counteract orthopaedic infections, while the local antibiotic delivery represents one option of the effective strategies to prevent and even to treat bone infections [13]. Although various delivery systems of local antibiotics have been attempted [14,15], the amount and rate of antibiotics release from this delivery system are still not satisfactory [5]. 

As a material able to locally convey antibiotics and alleviate bone bacterial infections [16], antibiotic-loaded hydrogel may represent a promising potential alternative to provide infection control related to orthopaedic devices [17,18]. The purpose of this study is to establish an effective slow-release system of antibiotics encapsulated within the transglutaminase (TGase) cross-linked gelatin-alginate hydrogel; we further test the efficacy of antibiotic-encapsulated hydrogel on the surface of titanium pin in prevention of peri-implant infections with a rat model. 

## 2. Materials and Methods

### 2.1. Purification of Transglutaminase (TGase)

First 4g unpurified TGase (ACTIVA TI, Ajinomoto, Tokyo, Japan) from Streptomyces spp. was completely dissolved in 40 mL phosphate buffer (20 mM phosphate and 2 mM ethylenediaminetetraacetic acid, pH 6.0), then mixed and spun for 24 h at 4 °C. The supernatant was harvested by centrifugation at 5000 rpm for 10 min twice. The TGase was purified y using (30K Da) Amicon^®^Ultra-15 Centrifugal Filter Units (Millipore, Merck, Burlington, MA, USA), centrifugation at 5000 rpm with buffer changed twice. Bradford protein essay (Bio-Rad, Hercules, CA, USA) was used to determine the final enzyme concentration by using bovine serum albumin (BSA) as standard. Briefly, the protein solution (standard and sample) were diluted till 10–100 µg protein at least in one assay tube. Then, mixed the solutions well after pipetting them and read the absorption at 590 nm by prewarmed spectrophotometer 5 min later. 

The crosslinking effect of purified TGase was verified by “two-syringe mixing” and the “inverted vial” method. Briefly, the gelatin/alginate (10% w/w ratio) mixture were crosslinked with three different con-centration of transglutaminase (TGase: 0, 0.5% and 1%). The gelation time and degree of crosslink between hydrogel at 3 different concentrations of transglutaminase (TGase) were visually observed.

### 2.2. Gelatin/Alginate/TGase Preparation

TGase was used to covalently cross-link gelatin (Type A, porcine, Sigma-Aldrich, St. Louis, MO, USA) and alginate (Sodium alginate, Merck KGaA, Darmstadt, Germany). Briefly, TGase was slowly added into gelatin/alginate (10% w/w ratio) mixture until a concentration of 25 mg/mL was reached. The mixture was allowed to react under room temperature for 18 h. All the samples were dehydrated in a graded ethanol solution (50%, 75%, 85%, and 95%, each for 5 min, and 100% three times for 10 min) before applying the critical-point drying (CPD) method, and were sputter-coated with gold to a thickness film before observation. Morphology of the resulting hydrogel was observed by scanning electron microscope (JSM-5600, JEOL Technics, Tokyo, Japan).

### 2.3. Degree of Crosslink

The ninhydrin test is a chemical testing method commonly used for verifying the presence of amino acids. Briefly, aliquot the reaction mixture into 20 μL aliquots in 0.5 mL Eppendorf tubes and 100 μL of 0.4% w/v ninhydrin in 80% DMSO/20% water mixture buffered at pH 7.5, and mix thoroughly. Incubate the samples at 85 °C for 15 min. The color of the samples should become blue. Cool the samples to room temperature, add 200 µL of water, and mix. The color of the samples should turn violet. Transfer 100 µL of each sample into the wells of a 96-well plate and measure the optical density (OD) at 570 nm using a microplate reader (Ultraviolet/Visible Spectrophotometer (UV-VIS), SpectraMax i3x, Molecular Devices, CA, USA), then quantify the free amine content through its relationship with OD value.

### 2.4. Drug Release Profile

Prepared gelatin/alginate/gentamicin(100mg/L) or vancomycin(100mg/L)/TGase hydrogel was submerged in phosphate-buffered saline (PB)S solution at 37 °C. Supernatant sample was collected at different time interval (0, 20, 40, …, 120 h) to evaluate the drug release capability for Gentamicin/Vancomycin. To detect gentamicin and vancomycin amount in samples, indirect competitive enzyme-linked immunosorbent assay (ELISA) for gentamicin or vancomycin was performed as previously reported [19]. Briefly, two specific competitive ELISA-assays were set-up to detect either gentamicin or vancomycin in protein-rich samples. An antibiotic-BSA hapten (10 ng coupled G-BSA, 1 µg V-BSA or 1.34 µg coupled G/V-BSA per well) was generated as a coatable antigen to the surface of a 96-wells ELISA plate and commercially available antibodies [50 microliter of diluted primary antibody (mouse anti-gentamicin monoclonal antibody (Abcam, USA), 7000× diluted in PBS/BSA; rabbit anti-vancomycin polyclonal antibody (Abd Serotec, UK), 5000× diluted in PBS/BSA)] were applied for downstream immunodetection. After incubation, the ELISA plate was washed 4 times and 100 microliter diluted secondary antibody (for gentamicin: rabbit anti mouse peroxidase (RAMPO, Dako, Denmark), 5000× diluted in PBS/BSA; for vancomycin: swine anti-rabbit peroxidase (SWARPO, Dako, Denmark), 2000x diluted in PBS/BSA), washed, and 100 µl 3,3′,5,5′-tetramethylbenzidine (TMB, Sigma-Aldrich, USA) was added to each well to allow chromogenic detection of bound secondary antibodies. After stopping the reaction, the absorbance at 450 nm was measured using an ELISA reader (MultiSkan FC, Thermo Scientific, Waltham, MA, USA). 

### 2.5. Cell Viability

Cell viability was evaluated with a LIVE/DEAD^®^ Violet Viability/Vitality Kit according to manufacturer’s instruction. Briefly, 5 × 10^4^ NIH 3T3 cell line (NIH 3T3 Cell Line murine; Merck KGaA, Darmstadt, Germany) were stained blue with calcium violet dye (ex/em = 400/452 ± 5 nm), dead cells were stained red with Aqua-fluorescent dye (ex/em = 400/526 ± 5 nm), and nucleus were stained blue using Hoechst 34580 (ex/em = 392/440 ± 5 nm). Images of cells were obtained using a confocal microscope (TCS SP5 II, Leica, Buffalo Grove, IL, USA) after staining. Cell viability was obtained from dividing dead cell count by total nucleus count.

### 2.6. Animal Model and Surgical Procedure

For each animal a fresh inoculum of a Methicillin-resistant *Staphylococcus aureus* (MRSA reference strain: ATCC 33591) was prepared by an overnight culture at 37 °C in brain-heart infusion (BHI) broth (Sigma-Aldrich, Inc., St. Louis, MO, USA). Cells were pelleted and washed twice in phosphate-buffered saline (PBS). Colony forming unit (CFU) counts were determined by serial dilution plating on blood agar. The final bacterial suspension in PBS consisted of 10^8^ CFU/50 μL. 

**Figure 1:**
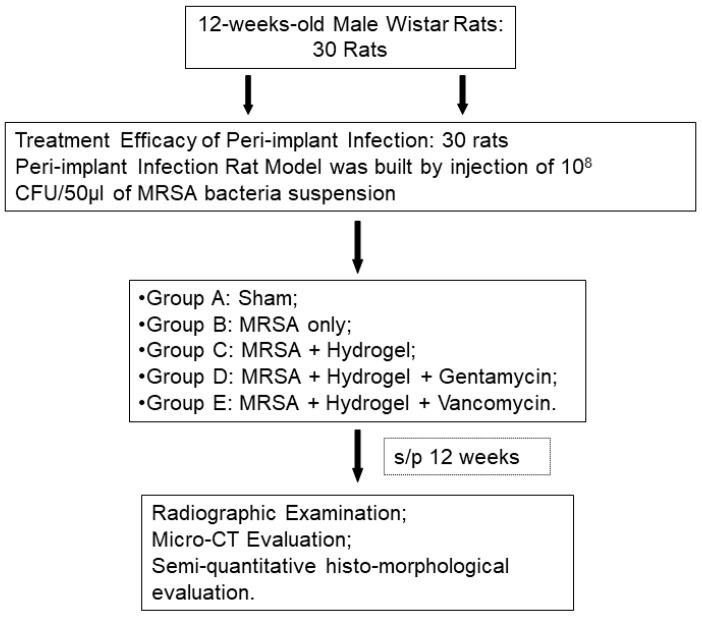
Flow chart of experimental design.

The experimental protocol was approved by the Institutional Animal Care and Use Committee of Medical College, National Taiwan University (Taipei, Taiwan). Thirty 12-week-old male Wistar rats (300 ± 20 g) were purchased from the Laboratory Animal Center (Figure 1), Medical College, National Taiwan University (Taipei, Taiwan) and acclimated under standard laboratory conditions at 22 ± 2 °C and 50 ± 10% humidity. Standard rat chow and water were available ad libitum during the acclimation period. Animals were anesthetized by Zoletil (15–18 mg/kg) and Rompun (0.05 mL/kg). Surgical interventions were performed under strict sterile conditions. The surgical area was shaved and disinfected with iodine, and a midline knee incision was made, followed by medial parapatellar arthrotomy and lateral patella subluxation to expose the knee joint. In this study, the peri-implant infections model was built by implanting a 0.7 mm titanium pin into the bone marrow cavity of the distal femur after injecting 10^8^ CFU/50 μL bacterial suspension of MRSA through 18 G needle; while in the sham-operated negative control rats, no metallic pin nor bacteria was implanted, only the injection of normal saline solution was undertaken. The arthrotomy was closed using non-resorbable sutures in an interrupted pattern and then the skin was closed with resorbable suture. Intramuscular injection of cefazoline (20 mg/kg, Purzer Pharmaceutical Co., Ltd., Taipei, Taiwan) was used for postoperative prophylaxis [20].

In the evaluation for the efficacy of gelatin/alginate/gentamicin or vancomycin/TGase hydrogel for peri-implant infections, different treatment modalities (total volume 0.5 cc) was implanted into the bone marrow cavity of distal femur by a metallic 23G needle. All of the 30 animals were divided into 5 groups (Table 1): Group A: sham (sterile saline solution only); Group B: infection positive control without treatment [MRSA only: 10^8^ CFU/50 μL bacterial suspension of Methicillin-Resistant *Staphylococcus Aureus* (MRSA)]; Group C: infection positive control with hydrogel treatment (MRSA+ Hydrogel); Group D: infection positive control with gentamicin-hydrogel treatment (MRSA+ Hydrogel+ Gentamicin: 20 mg/kg of body weight); Group E: infection positive control with vancomycin-hydrogel treatment (MRSA+ Hydrogel+ Vancomycin: 25 mg/kg/ kg of body weight) (Table 1). For groups C, D, and E, the hydrogels were injected into the marrow cavity just before the implantation of titanium pin. Animals from each group were sacrificed by overdose of pentobarbital at 12 weeks after surgery.

### 2.7. Radiographic Examination and Micro-CT (Computed Tomography) Evaluation

For radiographic evaluation, the soft tissue attached to the femur was removed, the rat femurs were examined by X-ray (Siemens, Germany). After radiologic examination, implants were removed and then assessed three dimensionally using Micro-CT (SkyScan 1176; Bruker Micro-CT, Kontich, Belgium) [21]. Datasets were reconstructed using CTvox 2.4 software for fast volumetric reconstruction, 2D/3D quantitative analysis and realistic 3D visualization. The tissue volume (TV: mm^3^), bone volume (BV: mm^3^) were recorded and then percent bone volume (BV/TV: %) was analyzed. After radiography, the femurs were decalcified and processed for H&E stain.

### 2.8. Histo-Morphological Analysis

After radiographic evaluation, the right-side specimens underwent decalcification, and then were embedded in paraffin. Paraffin-embedded sections (7 μm) were stained with hematoxylin and eosin for observational histology. An innovative approach to histopathological diagnostics and scoring system of osteomyelitis [Histopathological Osteomyelitis Evaluation Score (HOES)] with minor modification [22] was used for histo-morphological evaluation. The semi-quantitative analysis was performed by a blinded investigator.

### 2.9. Statistical Analysis

All results were presented as means ± standard deviation (SD). Statistical analysis was performed for all the quantitative results using analysis of variance (ANOVA), the post hoc analysis used was Tukey’s honestly significant difference. The overall analysis was performed by IBM Statistics SPSS 16.0 and the statistical significance in each test was set at *p* < 0.05.

## 3. Results

### 3.1. Degree of Crosslink

**Figure 2:**
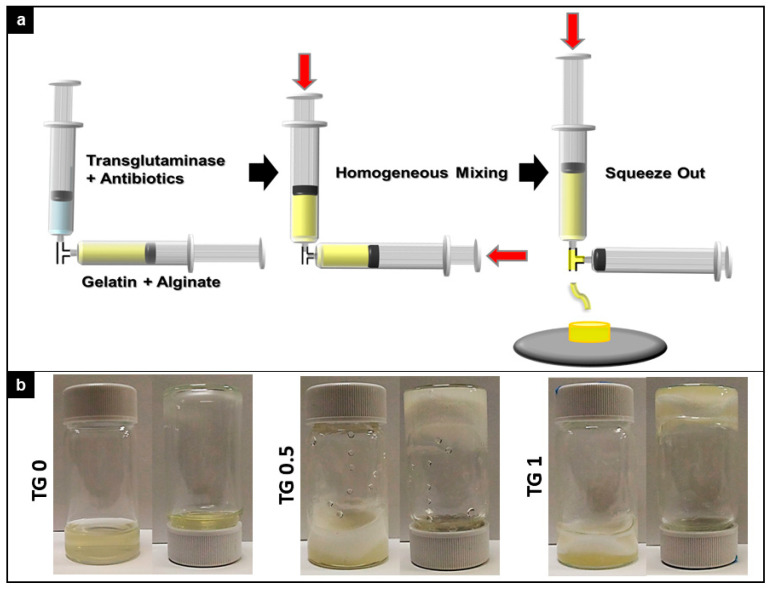
Preparation scheme of crosslink apparatus and degree of crosslink between three different concentration of transglutaminase (TGase) added. (**a**). Graphical representation of crosslink apparatus for the experiment. (**b**). Visual indication of degree of crosslink between three different concentration of transglutaminase (TGase) added. Both 0.5% and 1.0% TGase can effectively cross link the gelatin/alginate/vancomycin hydrogel.

As depicted in Figure 2a, gelatin and alginate were thoroughly mixed with transglutaminase (TGase) and antibiotics before squeezing out. A visual representation of the rigidity of resulting hydrogel can be seen in Figure 2b. Both 0.5% and 1.0% TGase can effectively cross link the gelatin/alginate/vancomycin hydrogel. TGase at 1% resulted in the most rigid hydrogel that stayed at the bottom of the container even when inverted.

**Figure 3:**
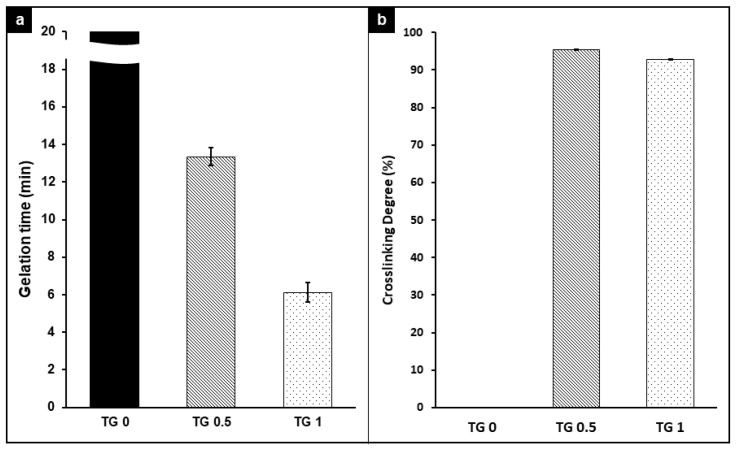
Gelation time (**a**) and degree of crosslink (**b**) between hydrogel with 3 different concentrations of transglutaminase (TGase) added. The gelation time decreased as TGase concentration increased, and the presence of TGase achieved over 90% crosslink for both 0.5% and 1.0%.

Gelation time and degree of crosslink can be seen at Figure 3a,b, respectively. TGase at 1% achieved the fastest gelation time at around 6 min, while without the presence of TGase resulted it over 20 min of gelation time. The percentage of crosslinking was achieved over 90% crosslink for both 0.5% and 1% TGase, while no TGase produced no crosslink.

**Figure 4:**
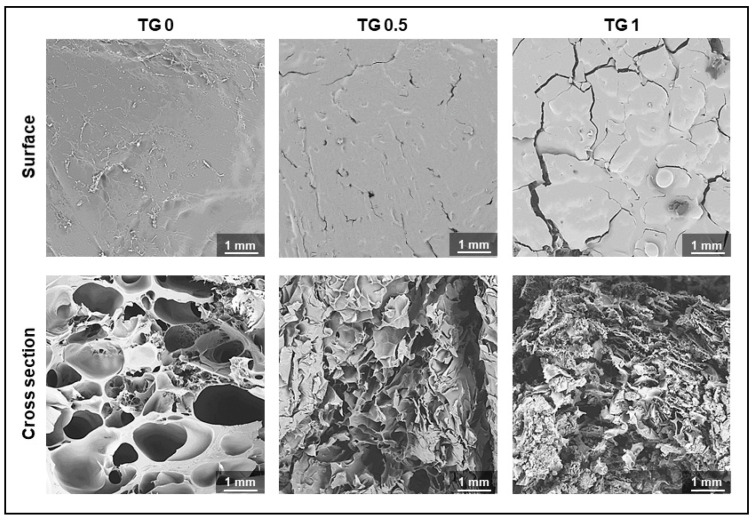
Electronic microscopic photos showing degree of crosslink of hydrogel with 3 different concentrations of transglutaminase (TGase) added. The surface becomes rougher and the cross section showed denser linkage as TGase concentration increase.

Figure 4 showed a visual representation of the surface and degree of crosslink of hydrogel under an electron microscope. As shown, the surface became rougher and the cross section showed denser linkage as TGase concentration increased.

### 3.2. Drug Release

**Figure 5:**
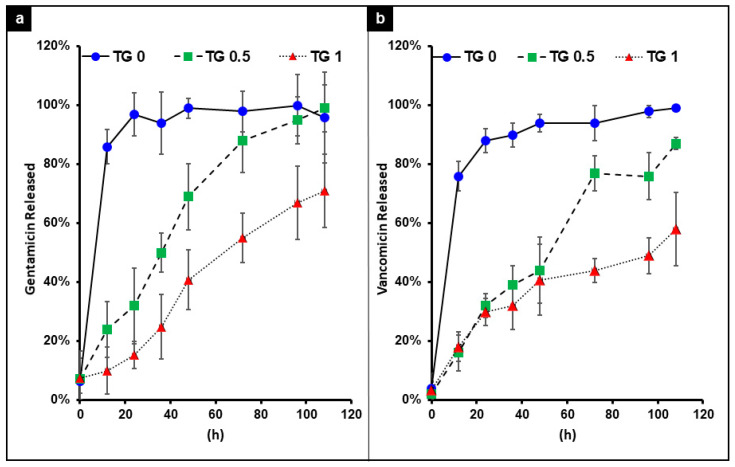
The release time of gentamicin (**a**) and vancomycin (**b**) encapsulated by hydrogel with 3 different concentrations of transglutaminase (TGase) added for crosslinking. With no TG the release of both drugs was near instantaneous. As the concentration of TG increases, a better slow release of the drug was achieved.

The release time for both antibiotics exhibit the same trend: the release of antibiotic slowed down with higher degree of TGase concentration. As shown in Figure 5, with no TGase the hydrogel achieved near 100% antibiotic release at around 20 min; while with TGase at the concentration of 0.5%, crosslink this was maintained possibly around 120 h; at the concentration of 1% TGase, antibiotic only partial released even after 120 h.

### 3.3. Cell Viability

**Figure 6:**
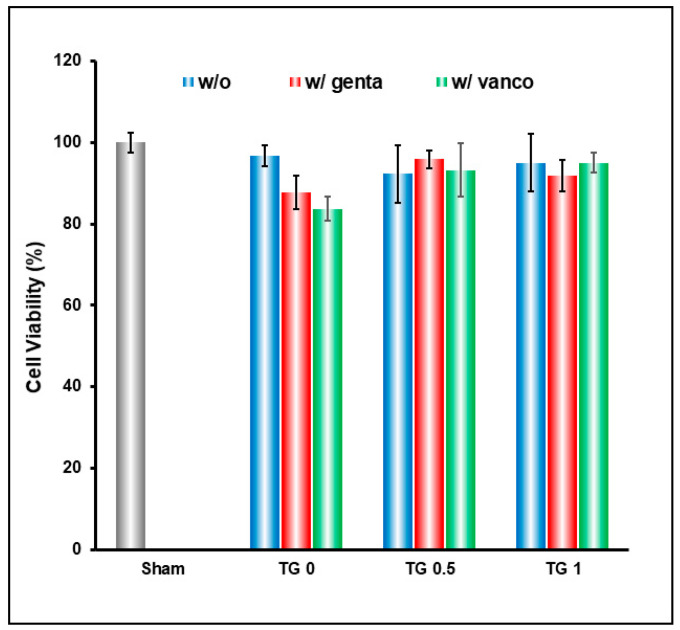
Cell viability result of hydrogel crosslinked with 3 different concentrations of transglutaminase (TGase), and encapsulating gentamicin and vancomycin. Without crosslink (TG0), the drug released was too rapid and resulted in slight decrease in cell viability; this is remedied by crosslinking the hydrogel to achieve slow release of drugs.

Without crosslink (TG-0), the drug released was too rapid which resulted in slight decrease in cell viability; this is remedied by crosslinking the hydrogel to achieve slow release of drugs (Figure 6).

### 3.4. In Vivo Animal Study

**Figure 7:**
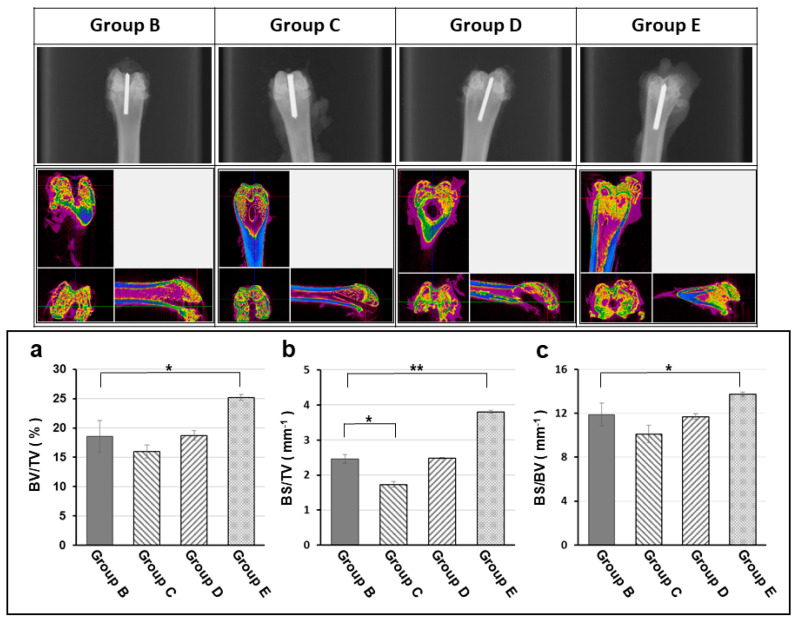
Radiographic, micro-CT (computed tomography) examination and bone volume change (**a**: Bone volume density = BV/TV; **b**: Bone surface density in total volume = BS/TV; **c**: Bone surface density = BS/BV) at the end of experiment (12 week). The X-ray images (upper row) and micro-CT scans (middle row) showing the radiographic evaluation for degree of osteomyelitis of each experimental group at the end of experiment (12 week). The peri-implant osteomyelitis-induced bone loss is significantly lower in the TGase cross-linked gelatin/alginate/vancomycin hydrogel group. Group B: titanium implant only; Group C: titanium implant with hydrogel; Group D: titanium implant with hydrogel and Gentamicin; Group E: titanium implant with Vancomycin. All implants were loaded with 10^8^ CFU/5 mL *Staphylococcus aureus* to promote infection. (BV: Bone Volume, TV: Total Volume, BS: Bone Surface; *: *p* < 0.05; **: *p* < 0.01)

The experimental animals were separated into 5 groups, each of 4 experimental groups receiving a titanium implant pretreated with different hydrogel/antibiotic combination, and were loaded with the same amount of 10^8^ CFU/50 μL MRSA to promote infection, as detailed in Table 1. At the end of the experiment, X-ray images of each experimental group were taken (Figure 7) while the micro-CT was examined to evaluate the bone recovery 12 weeks after surgery. There existed significant difference in bone volume areas among these 4 experimental groups. Group E (vancomycin treatment group) showed significant higher in bone volume areas relative to that of positive control group (B: MRSA only); in contrast, the bone volume had no significant difference within all the other groups (Figure 7). 

**Figure 8:**
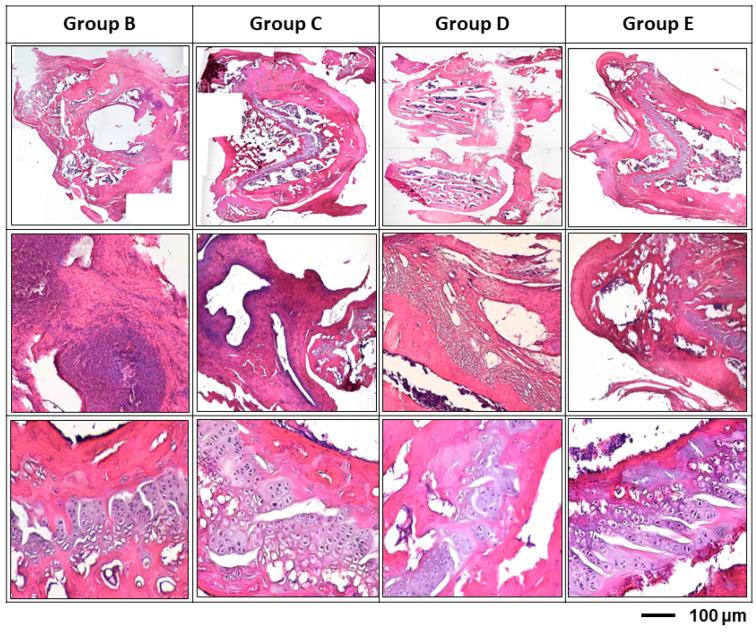
Histological examination of different treatment modalities for osteomyelitis rats after 12 weeks’ infection. After inoculation of *Staphylococcus aureus*, tissue necrosis with active inflammation and even biofilm formation was clearly demonstrated in all groups; while, the biofilm formation was found both the at the surface of the inserted pin and the inner surface of bony trabeculae. Group B: titanium implant only; Group C: titanium implant with hydrogel; Group D: titanium implant with hydrogel and gentamicin; Group E: titanium implant with vancomycin. All implants were loaded with 108 CFU/5 mL *Staphylococcus aureus* to promote infection.

Antibiotic-impregnated hydrogel can be effective in preventing peri-implant infections. As shown in Figure 8, the group E (vancomycin treatment group) had reduced the inflammation and provided more intact tissue structure. The noteworthy results were group B and group C; these 2 groups shown the similar results; there were significant tissue necrosis with active inflammation and even biofilm formation. In the semi-quantitative histo-morphological evaluation of osteomyelitis, group B showed significant inflammation, moderate bone regeneration and minimal intact bony structure; while group E (vancomycin treatment group) showed mild inflammatory cell infiltration and significant bone regeneration and more intact bony structure (Table 2).

## 4. Discussion

The continuously increasing demand for medical device implantations during surgical treatment represents a real clinical burden and cost for the modern healthcare system. An orthopaedic implant is an important therapeutic device and plays a key role in the current management of clinical orthopaedic conditions. Despite the significant advancements in biomaterials for orthopedics and surgical protocols, implant-related infections are still a challenging issue; the implant-related infection remains a leading cause of implant failure [5]. 

In a recent systemic review for animal models of orthopedic infections, due to the great variety of bacterial strains and inocula used; the information developed from these in vivo studies still cannot provide reproducible relevant information for a translational approach to humans [23]. In studies using a rabbit model of acute and chronic osteomyelitis, the rate of infection depended on the bacterial load, which means 70% for low grade (10^3^ CFU) and 100% for high grade of infection (10^4^ to 10^6^ CFU), respectively. Moreover, the grade of infection varied with the time set for the development of osteomyelitis with a mean at 3.4 weeks [24]. In a rat tibia model for induced osteomyelitis in the metaphysis, low-doses of bacterial loads (10^2^–10^3^ CFU of *Staphylococcus aureus*) were inoculated to develop a model of implant-related infections for better mimic clinical constellations [25]. Currently, most animal models use high bacterial loads (>10^4^ CFU) to provide high infection rates for investigation of peri-implant infection. On the other hand, the use of low amounts of bacteria may be more advantageous to mimic clinical constellation of surgery-related infections. In this study, the MRSA load used was 10^8^ CFU/50 μL with an incubation period for 12 weeks to ensure high infection rate and for long term evaluation of treatment efficacy of implant-related infections.

Although implant related infection is relatively unusual in orthopedic surgery; but when such infections occur, the consequences can be devastating. Successful treatment of implant-associated infection requires both effective surgical modalities and adequate antibiotics treatment supervised by clinical microbiologists to provide an optimal individualized strategy for each patient [26,27]. Towards the progression of infection, bacterial adhesion and formation of biofilm on the surface of the implant plays an important process [28]; but adequate treatment of the implant surface may potentially mitigate the risk of infection [29]. Although a number of promising anti-biofilm agents have been studied, but their effective role in current orthopaedic practice remains to be assessed. A local release system on the implant surface is an excellent idea as its local drug infusion and barrier effect on resisting biofilm formation which cause implant-related infections [30]. In order to tackle the implant-related infection, a sustained release antibacterial coated implant might support systemic antibiotic prophylaxis in preventing implant-associated infection [31].

To prevent bacterial colonization on the implants, various treatment modalities of antibiotics have been proposed; most of these modalities are truly powerful therapeutic tools, but only showing high local antibiotic concentrations over the short term [15]. An emerging approach to face the problem can be taken by encapsulating the antibiotic in a stable system to improve the drug delivery, localize the drug release at the site of action and to decrease the side effects [32]. Current technologies are still far from clinical application in the translational orthopaedic practice. Vancomycin is the first-line therapy for MRSA bacteraemia and infective endocarditis [33,34]. Theoretically, a fast-resorbable antibacterial-loaded hydrogel coating may offer efficacy toward preventing early bacterial colonization; a system of long-term release to deliver antibacterial compounds locally to provide local antibacterial and antibiofilm protection is mandatory in current orthopedic practice. As shown in this study, we expect the vancomycin-treatment group will show less infection-related bone destruction and better bone formation than that of the gentamicin treatment group.

Bones are important organs that provide structural support for the body. Injured bone is unable to act properly as a lever for muscle contraction, leading to further complications [35,36]. However, normal bone growth is a limited and slow process; bone cannot regenerate spontaneously if a critical-sized injury develops [37]. Large bone defects usually occur after implant loosening with major osteolysis, periprosthetic fractures with extensive osseous defects, or peri-implant infections and pseudarthrosis (non-union) [38]. Proper treatment of injured bone without the risk of pei-implant infection is, therefore, important for general well-being.

In the previous study of our institute, the biofilm formation was quite clear at 20 days after MSSA injection [20]. In this study, we explored the possibility of utilizing TGase cross-linked gelatin/alginate/antibiotics hydrogel to prevent complications of peri-implant infections. Our results demonstrated that both 0.5% and 1.0% TGase can effectively cross link the gelatin/alginate/antibiotics hydrogel and achieved over 90% crosslink with better slow release of the drug up to 120 h. Even after a high dose of MRSA inoculation of this study, the vancomycin treatment group still preserved relatively high bone volume, reduced the inflammation, significant bone regeneration, more intact bony structure and less biofilm formation. This is in line with previous report that vancomycin has minimal impact on both bone marrow-derived and proximal femur-derived mesenchymal stem cells in rats [39], while serving as an effective control of the *S. aureus* population [40,41]. This also led to an improved environment for which osteogenesis can occur.

Implant-related infection may be catastrophic and result in poor functional outcome, chronic osteomyelitis, implant failure or even sepsis and death. Prophylactic use of antibiotics is indicated when the risk of infection or the severity of possible complications outweigh the disadvantages of the regular use of antibiotics [42,43]. In this study, we developed a relatively slow-release resorbable hydrogel to deliver antibiotics and anti-biofilm compounds locally to reduce or prevent bacterial colonization and biofilm formation on the surface of biomaterials commonly used in orthopaedic surgery; the hydrogel is a feasible of intraoperative manipulation and possess the capability to resist press-fit intramedullary implant insertion for future clinical translation. For experimental animals with risk factors for primary wound infection, presurgical antibiotics are indicated for such surgical procedures. Cefazolin, frequently administered systemically for prophylaxis in a variety of surgical procedures, has been shown to be effective in treating methicillin-susceptible *Staphylococcus aureus* (MSSA) but does not work in cases of methicillin-resistant *Staphylococcus aureus* (MRSA) [44]. Gentamicin is active against a wide range of bacterial infections, mostly Gram-negative bacteria and the Gram-positive *Staphylococcus*. Vancomycin is recommended intravenously as a treatment of choice for complicated infections caused by methicillin-resistant *Staphylococcus aureus* [45]. However, in this study, intramuscular injection of cefazoline was used for postoperative prophylaxis; although, cefazolin may not interfere with the interpretation of our result, this still should be considered as a confounding factor in terms of the treatment effect of our study. 

## 5. Conclusions

State-of-the-art strategies to reduce the risk of implant-related infection are yet to develop as routine in orthopaedic clinical practice [46]. In this study, we developed a slow-release resorbable hydrogel to deliver antibiotics locally to prevent bacterial colonization on the surface of implants commonly used in orthopaedic surgery; the hydrogel is a feasible of intraoperative manipulation and possess the capability to resist press-fit intramedullary implant insertion. However, the remaining challenges will continue to be a topic of research, to confer different potent antibacterial medications with good biocompatibility and fulfill the safety, practical and economic criteria for clinical translation.

## Figures and Tables

**Table 1:** Experimental treatment-design of peri-implant osteomyelitis.

Group	A	B	C	D	E
Content	Sham	MRSA Only	MRSA + Hydrogel	MRSA + Hydrogel + Gentamicin	MRSA + Hydrogel + Vancomycin
*Staphy. Aureus* (10^8^ CFU/50 μL) + Titanium pin	−	+	+	+	+
Hydrogel	−	−	+	+	+
Gentamicin	−	−	−	+	−
Vancomycin	−	−	−	−	+

**Table 2:** T Semi-quantitative histo-morphological evaluation of osteomyelitis by histopathological osteomyelitis evaluation scoring system [Histopathological Osteomyelitis Evaluation Score (HOES)].

Group	B	C	D	E
Evaluation	MRSA Only	MRSA + Hydrogel	MRSA + Hydrogel + Gentamicin	MRSA + Hydrogel + Vancomycin
Inflammation	+ + + +	+ + +	+ +	+
Regeneration	+ +	+ +	+ + +	+ + + +
Intact Structure	+	+ +	+ + +	+ + + +

## References

[1] Chouirfa H., Bouloussa H., Migonney V., Falentin-Daudré C. (2019). Review of titanium surface modification techniques and coatings for antibacterial applications. Acta Biomater..

[2] Drago L., Clerici P., Morelli I., Ashok J., Benzakour T., Bozhkova S.A., Ali-Zadeh C., Del Sel H., Sharma H., Peel T.N. (2019). The World Association against Infection in Orthopaedics and Trauma (WAIOT) procedures for Microbiological Sampling and Processing for Periprosthetic Joint Infections (PJIs) and other Implant-Related Infections. J. Clin. Med..

[3] Zimmerli W., Sendi P. (2017). Orthopaedic biofilm infections. APMIS.

[4] Wang Y., Cheng L.I., Helfer D.R., Ashbaugh A.G., Miller R.J., Tzomides A.J., Thompson J.M., Ortines R.V., Tsai A.S., Liu H. (2017). Mouse model of hematogenous implant-related Staphylococcus aureus biofilm infection reveals therapeutic targets. Proc. Natl. Acad. Sci. USA.

[5] Wang M., Tang T. (2019). Surface treatment strategies to combat implant-related infection from the beginning. J. Orthop. Translat..

[6] Metsemakers W.-J., Kuehl R., Moriarty T., Richards G.R., Verhofstad M., Borens O., Kates S., Morgenstern M. (2018). Infection after fracture fixation: Current surgical and microbiological concepts. Injury.

[7] Zimmerli W., Moser C. (2012). Pathogenesis and treatment concepts of orthopaedic biofilm infections. FEMS Immunol. Med. Microbiol..

[8] Liu S., Ji X.-X., Zhu J.-F. (2020). Recent Progress on the Synthesis and Biomedical Properties of Natural Biopolymer Composites. Curr. Med. Chem..

[9] Yan M., Chen T., Zhang S., Lu T., Sun X. (2020). A core-shell structured alginate hydrogel beads with tunable thickness of Carboxymethyl cellulose coating for pH responsive drug delivery. J. Biomater. Sci. Polym. Ed..

[10] Daly A.C., E Critchley S., Rencsok E.M., Kelly D.J. (2016). A comparison of different bioinks for 3D bioprinting of fibrocartilage and hyaline cartilage. Biofabrication.

[11] Duarte L., Matte C.R., Bizarro C.V., Ayub M.A.Z. (2020). Transglutaminases: Part I—origins, sources, and biotechnological characteristics. World J. Microbiol. Biotechnol..

[12] Dell’Olmo E., Gaglione R., Arciello A., Piccoli R., Cafaro V., Di Maro A., Ragucci S., Porta R., Giosafatto C.V.L. (2021). Transglutaminase-mediated crosslinking of a host defence peptide derived from human apolipoprotein B and its effect on the peptide antimicrobial activity. Biochim. Biophys. Acta (BBA) Gen. Subj..

[13] Giavaresi G., Minelli E.B., Sartori M., Benini A., Della Bora T., Sambri V., Gaibani P., Borsari V., Salamanna F., Martini L. (2012). Microbiological and pharmacological tests on new antibiotic-loaded PMMA-based composites for the treatment of osteomyelitis. J. Orthop. Res..

[14] Neut D., Dijkstra R.J., Thompson J.I., Kavanagh C., van der Mei H.C., Busscher H.J. (2015). A biodegradable gentamicin-hydroxyapatite-coating for infection prophylaxis in cementless hip prostheses. Eur. Cell Mater..

[15] Drago L., Boot W., Dimas K., Malizos K., Hänsch G.M., Stuyck J., Gawlitta D., Romanò C.L. (2014). Does Implant Coating With Antibacterial-Loaded Hydrogel Reduce Bacterial Colonization and Biofilm Formation in Vitro?. Clin. Orthop. Relat. Res..

[16] Changez M., Koul V., Dinda A.K. (2005). Efficacy of antibiotics-loaded interpenetrating network (IPNs) hydrogel based on poly(acrylic acid) and gelatin for treatment of experimental osteomyelitis: In vivo study. Biomaterials.

[17] Zagra L., Gallazzi E., Romanò D., Scarponi S., Romanò C. (2019). Two-stage cementless hip revision for peri-prosthetic infection with an antibacterial hydrogel coating: Results of a comparative series. Int. Orthop..

[18] Boot W., Vogely H., Nikkels P., Pouran B., Van Rijen M., Ekkelenkamp M., Hänsch G., Dhert W., Gawlitta D. (2020). Prophylaxis of implant-related infections by local release of vancomycin from a hydrogel in rabbits. Eur. Cell Mater..

[19] Odekerken J.C., Logister D.M., Assabre L., Arts J.J., Walenkamp G.H., Welting T.J. (2015). ELISA-based detection of gentamicin and vancomycin in protein-containing samples. Springerplus.

[20] Fang C.-H., Tsai P.-I., Huang S.-W., Sun J.-S., Chang J.Z.-C., Shen H.-H., Chen S.-Y., Lin F.-H., Hsu L.-T., Chen Y.-C. (2017). Magnetic hyperthermia enhance the treatment efficacy of peri-implant osteomyelitis. BMC Infect. Dis..

[21] Al-Hezaimi K., Ramalingam S., Al-Askar M., ArRejaie A.S., Nooh N., Jawad F., Aldahmash A., Atteya M., Wang C.Y. (2016). Real-time-guided bone regeneration around standardized critical size calvarial defects using bone marrow-derived mesenchymal stem cells and collagen membrane with and without using tricalcium phosphate: An in vivo micro-computed tomographic and histologic experiment in rats. Int. J. Oral Sci..

[22] Tiemann A., Hofmann G.O., Krukemeyer M.G., Krenn V., Langwald S. (2014). Histopathological Osteomyelitis Evaluation Score (HOES)—An innovative approach to histopathological diagnostics and scoring of osteomyelitis. GMS Interdiscip. Plast. Reconstr. Surg. DGPW.

[23] Lovati A.B., Bottagisio M., De Vecchi E., Gallazzi E., Drago L. (2017). Animal Models of Implant-Related Low-Grade Infections. A Twenty-Year Review. Adv. Exp. Med. Biol..

[24] Bottagisio M., Coman C., Lovati A.B. (2019). Animal models of orthopaedic infections. A review of rabbit models used to induce long bone bacterial infections. J. Med. Microbiol..

[25] Harrasser N., Gorkotte J., Obermeier A., Feihl S., Straub M., Slotta-Huspenina J., Von Eisenhart-Rothe R., Moser W., Gruner P., De Wild M. (2016). A new model of implant-related osteomyelitis in the metaphysis of rat tibiae. BMC Musculoskelet Disord..

[26] Edwards C., Sheppard N.N. (2018). Prevention, Diagnosis, and Treatment of Implant Infection in the Distal Upper Extremity. J. Hand. Surg. Am..

[27] Drampalos E., Mohammad H.R., Pillai A. (2020). Augmented debridement for implant related chronic osteomyelitis with an absorbable, gentamycin loaded calcium sulfate/hydroxyapatite biocomposite. J. Orthop..

[28] Moormeier D.E., Bayles K.W. (2017). Staphylococcus aureus biofilm: A complex developmental organism. Mol. Microbiol..

[29] ter Boo G.J., Grijpma D.W., Moriarty T.F., Richards R.G., Eglin D. (2015). Antimicrobial delivery systems for local infection prophylaxis in orthopedic- and trauma surgery. Biomaterials.

[30] Pan C., Zhou Z., Yu X. (2018). Coatings as the useful drug delivery system for the prevention of implant-related infections. J. Orthop. Surg. Res..

[31] Han J., Yang Y., Lu J., Wang C., Xie Y., Zheng X., Yao Z., Zhang C. (2017). Sustained release vancomycin-coated titanium alloy using a novel electrostatic dry powder coating technique may be a potential strategy to reduce implant-related infection. Biosci. Trends..

[32] Cusumano J.A., Klinker K.P., Huttner A., Luther M.K., Roberts J.A., Laplante K.L. (2020). Towards precision medicine: Therapeutic drug monitoring–guided dosing of vancomycin and β-lactam antibiotics to maximize effectiveness and minimize toxicity. Am. J. Health Syst. Pharm..

[33] Mermel L.A., Allon M., Bouza E., Craven D.E., Flynn P., O’Grady N.P., Raad I.I., Rijnders B.J.A., Sherertz R.J., Warren D.K. (2009). Clinical practice guidelines for the diagnosis and management of intravascular catheter-related infection: 2009 Update by the Infectious Diseases Society of America. Clin. Infect Dis..

[34] Turner N.A., Sharma-Kuinkel B.K., Maskarinec S.A., Eichenberger E.M., Shah P.P., Carugati M., Holland T.L., Fowler V.G. (2019). Methicillin-resistant Staphylococcus aureus: An overview of basic and clinical research. Nat. Rev. Microbiol..

[35] Clarke B.L. (2008). Normal Bone Anatomy and Physiology. Clin. J. Am. Soc. Nephrol..

[36] Buck D.W., Dumanian G.A. (2012). Bone biology and physiology: Part II. Clinical correlates. Plast. Reconstr. Surg..

[37] Keating J.F., Simpson A.H.R.W., Robinson C.M. (2005). The management of fractures with bone loss. Bone Joint J..

[38] Zajonz D., Birke U., Ghanem M., Prietzel T., Josten C., Roth A., Fakler J.K.M. (2017). Silver-coated modular Megaendoprostheses in salvage revision arthroplasty after periimplant infection with extensive bone loss—a pilot study of 34 patients. BMC Musculoskelet Disord..

[39] Booysen E., Gijsen H.S.-V., Deane S.M., Ferris W., Dicks L.M.T. (2018). The Effect of Vancomycin on the Viability and Osteogenic Potential of Bone-Derived Mesenchymal Stem Cells. Probiotics Antimicrob. Proteins.

[40] Zhang Y., Shen L., Wang P., Xi W., Yu Z., Huang X., Wang X., Shou D. (2019). Treatment with Vancomycin Loaded Calcium Sulphate and Autogenous Bone in an Improved Rabbit Model of Bone Infection. J. Vis. Exp..

[41] Bishop A.R., Kim S., Squire M.W., E Rose W., Ploeg H.-L. (2018). Vancomycin elution, activity and impact on mechanical properties when added to orthopedic bone cement. J. Mech. Behav. Biomed. Mater..

[42] van den Bogaard A.E. (1984). Antibiotic prophylaxis in small-animal surgery. Tijdschr Diergeneeskd..

[43] van den Bogaard A.E., Weidema W.F. (1985). Antimicrobial prophylaxis in veterinary surgery. J. Am. Vet. Med. Assoc..

[44] Fallon M.T., Shafer W., Jacob E. (1999). Use of Cefazolin Microspheres to Treat Localized Methicillin-Resistant Staphylococcus aureus Infections in Rats. J. Surg. Res..

[45] Ceccato A., Di Giannatale P., Nogas S., Torres A. (2021). Safety considerations of current drug treatment strategies for nosocomial pneumonia. Expert Opin. Drug Saf..

[46] Tobin E.J. (2017). Recent coating developments for combination devices in orthopedic and dental applications: A literature review. Adv. Drug Deliv. Rev..

